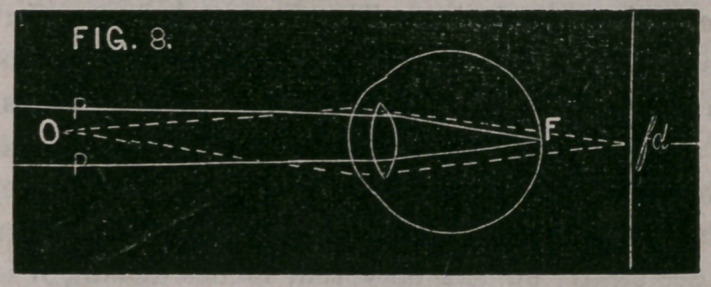# An Introduction to the Study of the Optical Defects of the Eye and Their Treatment by the Scientific Use of Spectacles

**Published:** 1866-02

**Authors:** A. M. Rosebrugh

**Affiliations:** Toronto


BUFFALO

Medical and Surgical Journal.

VOL. V. FEBRUARY, 1866. No. 7.

### Art. 1.—AN INTRODUCTION TO THE STUDY OF THE OPTICAL DEFECTS OF THE EYE AND THEIR TREATMENT BY THE SCIENTIFIC USE OF SPECTACLES.

---

BY A. M. ROSEBRUGH, M.D., TORONTO.

---

(The following pages were written as an introduction to a course of lectures on the diseases of the eye.)

In their preparation, I must here acknowledge my indebtedness to the elaborate works of Mr. J. Z. Laurence and Mr. J. Soelberg Wells, of London, and especially to the very comprehensive treatise of Professor Donders, of Utrecht, published in 1864 by the New Sydenham Society.

### Chapter I.—Optical Considerations.

The eye is pre-eminently an optical instrument, and the phenomena of vision all depend upon the laws of optics. Hence, a knowledge of some, at least, of the elementary principles of light is essential to a correct appreciation of the physiology of the eye. The diagnosing of optical defects of the eye,—long and short sight, &c. &c., and their treatment with the scientific use of spectacles, require some knowledge of the laws of refraction, and the properties of convex and concave lenses.

The philosophy of the ophthalmoscope can hardly be understood unless the principles of both refraction and reflection are thoroughly mastered.

You will therefore, I hope, not consider the time ill spent if, before proceeding with the investigation of diseases of the eye—you review with me some of the elementary principles of optics which lie at the foundation of all ophthalmic science.

The *nature* of light is not known. I can no more tell you what light is, than your professor of physiology can tell yon what life is. We know that the sun shines, but how it shines we cannot tell.

"Two different theories have been advanced of the more intimate nature of light." "One, the *Newtonian* (*corpuscular*) conceives that each luminous point is constantly giving off a succession of luminous corpuscles which follow each other in uninterrupted succession on an imaginary line or axis like a string of beads on a rigid thread."

The *undulatory* theory (Christian Huychens') on the other hand considers space as pervaded by a subtle gaseous fluid or ether; that luminous bodies have the power of communicating to this ether a wave motion which affects the retina the same as vibrations of the air affect the auditory nerve.

Sir John Herschel, speaking of the great ingenuity of the undulatory theory says, "if it is not true it deserves to be."

The sun is the great natural source of light; as it shines by its own light it is called *self-luminous*. The fixed stars are also self-luminous; so is a lighted lamp and bodies in a state of ignition. But most bodies by which we are surrounded, are seen only by reflected light. The light from an object seen by moonlight is reflected twice before it reaches the eye. The moon reflects the light from the sun, and the object, the light which it receives from the moon.

Every luminous object gives off, or radiates, in every direction, an infinite number of straight lines of light. Each of these lines taken alone is called a *ray* of light. A bundle of rays is called a *beam* of light when the rays run *parellel* to each other. When the rays *diverge* from a luminous point or are made to *converge* to a focus they are called a *pencil* of rays, thus :

Fig. 1 represents a pencil of rays diverging from a flame F, after passing a convex lens they are rendered parallel and these parallel rays passing the second convex lens B, the rays are converged to the point (focus) P.

The parallel rays may be called a *parallel* pencil ; the diverging rays a *divergent* pencil, and the convergent rays a *convergent* pencil. The point where rays of light meet is called the *focal* point or simply a *focus*.

Strictly speaking, there is no such thing in nature as parallel rays ; the nearest approach we have to it are the rays of light we receive from the sun and the fixed stars. Practically, for our purpose however, we may consider rays of light parallel that are received by the pupil of the eye from objects that are twenty feet distant or any distance greater than that. Pencils of light from objects less than twenty feet distant are more decidedly divergent.

A good illustration of a divergent pencil can be obtained from a lighted lamp or candle in a dark room. If a piece of card board, with a small circular opening in it, be held near the lamp, you will have, upon the opposite wall, an illuminated spot of the same shape as the opening in the card, but very much larger.

This will prove not only that the rays *diverge*, but also that the rays proceed in straight lines.*

*Convex lenses :*—We shall now proceed to the consideration of convex lenses, which, for our purpose, is the most important part of the subject. Lenses are made of various transparent substances as amber, alum, quartz, glass, diamond, and even of ice. Those in ordinary use are made of glass. When the two surfaces of a convex lens have the same degree of curvature, the lens is said to be equi-convex. When one of the surfaces is flat or plane, the lens is called a plano-convex lens. Glass spectacles used by old persons for reading, &c., are commonly made double convex.

In order to simplify the subject as much as possible, let us confine our attention to lenses that are equi-convex.

In fig. 2 let A be the centre of the circle B, C, D, of which A, B, is the radius, and let E be the centre of the circle F, G, H, of which the radius E, F, is equal to the radius A, B. The circle F, G, H, will be equal to the circle B, C, D. The part D,H, common to both circles, represent a section of an equi-convex lens. The line A,E, is called the *axis* of the lens, and the line D, H is called the *diameter*. The centre of the diameter (where it is intersected by the axis) is the optical centre of the lens.

Reading glasses, and burning glasses, are examples of a double convex lens. Many of you have, doubtless, seen the experiment of

(* Convergent pencils of light do not exist in nature. Parallel pencils or divergent pencils of rays can be rendered convergent by means of a convex lens. Thus in fig. 1, the rays diverging from F, are made to *converge* to P by the convex lenses, A. and B.)

setting fire to wood, paper, &c., by means of a burning or sun glass. The explanation of this is simply that the convex lens possesses the property of converging a portion of the sun's rays to a point called the focus.

In Fig. 3, P, P, represent a pencil of parallel rays converged to a focus at F by means of the double convex lens, L.

The focus for parallel rays is called the *principal* focus. It is always the same distance from the optical centre in the same lens. The length of the focus for parallel rays is, in equi-convex lenses, equal to the length of the radius of curvature.

The shorter the focus, the greater is the "power" or "strength" of the lens. A lens that can bring parallel rays to a focus at a distance of one inch from the optical centre of the lens, would be called a *one inch* lens. Another lens whose focus is two inches from the optical centre, is called a *two inch* lens, and so on. Convex lenses therefore receive their names according to the number of inches, or fraction of an inch, the principal focus is distant from the centre of the lens. The strongest lenses used for spectacles are what are called cataract glasses; they are worn by patients who have had their crystaline lenses removed. Their strength ranges from 2 to 4 inches focal length. The weakest spectacles that are ordinarily used have a focus of 36 inches. Convex lenses having a focus of 36 inches do not enlarge the letters of a book at the ordinary reading distance.

Let us now see what practical application we can make of this principle of convex lenses.

Supposing that a person accustomed to using convex spectacles, gets one of the glasses broken, and applies to you to learn the strength of the glass that would be necessary to replace the broken one, or in other words—to learn the strength of the glass that is still whole. How would you proceed? One method is to use the lens as a sun glass, and ascertain by measurement, how far from the glass, the sun's rays are brought to a focus. If you find, for instance, that the focus is 10 inches from the lens, you will have ascertained that the person has been wearing glasses of 10 inch focus, or as they are sometimes called No. 10 convex, or simply + 10 (plus 10).

The method, however, that is usually adopted, depends upon a property of convex lenses that will be more fully explained further on.

If, for instance, you hold up a 10 inch convex lens at a distance of 10 inches from a white wall—the wall being about 20 feet from an open window, opposite—there will appear, behind the lens, upon the wall, an inverted, miniature picture of the window, and trees or buildings, &c., in front of the window. If the lens be held at a greater or less distance from the wall than the focal length of the lens, the inverted picture will be indistinct. Measuring the distance therefore that the lens must be held from the wall, to produce the sharpest picture, will give the focal length of the lens.

Suppose, now, that we bring the lens to within, say 5 feet of the window, and hold a sheet of white paper at the principal focal distance behind the lens, viz., at ten inches, we will find a change in the inverted picture, there will still appear distant buildings, trees, &c. but the sash of the window will be very indistinct. If, however, we move the sheet of paper 12 inches from the lens—that is, two inches farther from the lens, we will again see the image of the sash but scarcely any trace of the buildings, trees, &c. This experiment is an illustration of the fact that the nearer an object approaches the front of a convex lens, the farther will be its image behind the lens ; thus, when an object is 5 feet or rather 60 inches from the front of a 10 inch convex lens, the inverted image is found to be 12 inches behind the lens ; when 30 inches, it will be 15 in. ; when 20, that is, double the length of the focus, the image will be double the length of the focus behind the lens ; viz., 20 inches ; when 15 inches, the image behind the lens will be removed to 30 inches. As the object approaches the principal focal distance of the lens the image recedes much more rapidly ; thus, when at 12 inches, the image will be 60 inches ; when at 11, the image will be 110 inches behind the lens. When however we bring the object to within 10 inches of the lens—that is, at its principal focus, there will be no image formed behind the lens, as the rays after passing the lens will be parallel.

(I would strongly urge you, gentlemen, to perform all these experiments for yourselves, as in that way only can you become familiar with these important principles. These latter experiments can be performed best in a dark room—taking for an object the flame of a lamp or candle).

From the above we can easily understand the principle, 1st, that the *less* divergent the rays of a pencil (that is, the nearer they approach parallel rays,) incident or falling upon a convex lens, the nearer will the focus of the convergent pencil be to the principal focus of the lens. 2nd. The *more* divergent the incident pencil, the less convergent (the more nearly parallel) will be the refracted pencil, and the more distant will its focus be from the principal focus of the lens.

Questions of the following nature very often arise in optics, viz., the length of the principal focus of a convex lens being given, and the distance a certain object is in front of it;—to find how far behind the lens will be the inverted image of the object. Or to express it more technically, the length of the principal focus of a convex lens being given and the length of the divergent incident pencil, to find the length of the focus of convergent refracted pencil. Thus: Suppose you had the following question: A 10 inch lens is 60 inches from an object; how far behind the lens will be the inverted image?

This could be solved immediately, by actual trial, and measurement, but this is not always practical.

The rule given in some text books on optics is as follows: multiply the length of the divergent incident pencil, that is, the distance the object is from the lens, by the focal length of the lens, and divide by the difference; thus: $60 \times 10 = 600$, $60 - 10 = 50$, 600 divided by $50 = 12$; or $\frac{60 \times 10}{60 - 10} = \frac{600}{50} = 12 =$ the distance behind the lens.

There is another property of convex lenses which I must not omit to mention; namely, what is called it magnifying power.

When a convex lens is placed between the eye and an object,—the object being at a less distance from the lens than its principle focus, the object will appear enlarged or magnified. The shorter the focus of the lens, the greater is its magnifying power. Thus, a 4 inch lens has a greater magnifying power than an 8 inch lens; a 2 inch lens greater than a 4, and a 1 inch greater than a 2 inch lens. The 1 inch lens has, in fact, double the magnifying power of a 2 inch lens; a 2, double that of 4 inch; a 4 inch, double that of an 8 inch, &c.

The "power" of a lens is therefore inversely proportional to its focal length. For this reason a different form is used in expressing the "power" or strength of a lens. A 1 inch lens is taken as unity, and as a 2 inch lens is just half the strength, it is simply expresed $\frac{1}{2}$, and as a 3 inch lens has just one-third the strength of a 1 inch, it is written $\frac{1}{3}$; a 4 inch is $\frac{1}{4}$ &c. We will find that this nomenclature is not only very convenient, but scientifically correct.

For example, suppose we have two lenses of 4 inch focus each, and we wish to know their combined "power" when used as one lens; we simply add their reciprocals thus $\frac{1}{4}+\frac{1}{4}=\frac{2}{4}=\frac{1}{2}$. The two lenses have, therefore, the magnifying power of $\frac{1}{2}$, which is the reciprocal of 2, and are consequently, together, equal to a 2 inch lens, which can be proved by actual measurement. Again, suppose we have a 6 inch lens, and a 12 inch lens, and we wish to know their combined strength, $\frac{1}{6}+\frac{1}{12}=\frac{3}{12}=\frac{1}{4}$ which represents the power of a 4 inch lens; the 6 and the 12 inch lenses taken together being equal to one lens having a focus of 4 inches.

To save repetition, I may here state that when a *concave* lens enters into combination with a *convex* lens, it has a neutralizing effect upon the convex lens. If we have a convex 6 and a concave 6 the one would neutralize the other,—thus $\frac{1}{6}-\frac{1}{6}=0$. But if the convex lens has the higher power, the concave lens simply weakens it—that is, lengthens its focus—thus, if we have a convex 6 and a concave 9 the result will be $\frac{1}{6}-\frac{1}{9}=\frac{3}{18}-\frac{2}{18}=\frac{1}{18}$, which represents the strength of one lens having a focus of 18 inches. If, however, the concave lens has the higher "power" it will simply be weakened by the concave lens,—the combination will be equal to a concave lens having a lower "power," or a longer focus than the concave lens taken,—thus reversing the last example, suppose we have a *concave* 6 and a *convex* 9, we will then have $-\frac{1}{6}+\frac{1}{9}$ or simply $\frac{1}{9}-\frac{1}{6}=\frac{2}{18}-\frac{3}{18}=-\frac{1}{18}$, which represents the strength of a *concave* lens having a focus of 18 inches.

This fractional nomenclature (taking 1 for numerator and the focal length of the lens for denominator) will assist us also in understanding the principle of the formation of images at different distances behind a convex lens, according to the distance of objects in front of it.

Let me remind you that when an object, for instance the flame of a candle, is placed in the focus of a convex lens, the diverging rays of light from the object are rendered parallel by the lens. Thus, a lens having a focus of 20 inches will render parallel pencils of light diverging from an object 20 inches from the lens. Bearing this in mind let us again try the solution of the following question, propounded not long since, viz. :—When an object is 60 inches in front of a 10 inch convex lens, how far behind the lens will be the inverted image of the object? Or, to express it differently, when a divergent pencil of light emanates from a point 60 inches from a 10 inch convex lens, at what distance behind the lens will the pencil be converged to a focus ?

Now, we know that a lens of 60 inches focus, placed in the position of the 10 inch lens, would render the rays parallel that fall upon it from the object 60 inches distant. Were it possible, therefore, to divide the 10 inch lens into two lenses, one having a focus of 60 inches to render the rays parallel, the remaining portion would bring these parallel rays to a focus at its principle focus. Deducting then $\frac{1}{60}$ from $\frac{1}{10}$ will give the strength of the remaining portion of the lens $\frac{1}{10}-\frac{1}{60}=\frac{5}{60}=\frac{1}{12}$; the two parts then $\frac{1}{60}$ and $\frac{1}{12}$ are equal to the one lens $\frac{1}{10}$. And, as the $\frac{1}{60}$ will render the rays parallel from the object 60 inches distant, and these parallel rays falling upon the other part $\frac{1}{12}$, they will be brought to a focus at the principle focus of this part, viz : at 12 inches from the lens. Let us illustrate this with another example. Suppose that an object is 30 inches in front of a convex lens of 10 inch focus, and we wish to know how far behind the lens will be the focus of a pencil of rays diverging from a point in the object. We will have $\frac{1}{15}-\frac{1}{30}=\frac{2}{30}=\frac{1}{15}$; this $\frac{1}{15}$ represents the power of a 15 inch lens, which we know will bring the parallel rays to a focus at 15 inches behind the lens.

Fig. 4 illustrates this ; O represents an object 30 inches from a ten inch convex lens, the lens supposed to be divided into two parts, one having a focus of 30 inches, and the other a focus of 15 inches. The 30 inch lens refracts the rays of the divergent pencil d, d, d, d, so as to render them parallel, as shown at P, P, P, P, P. These parallel rays, meeting the 15 inch lens, are again refracted and are converged to a focus at F, which is the principle focus of the lens, viz., at 15 inches.

Fig. 1, page 3, represents a 10 inch lens, at a distance of 20 inches from an object, F. The lens is supposed to be divided into two equal parts, of 20 inch focus each: the first half renders the diverging pencil parallel, and the second half converges the parallel pencil to a focus, at 20 inches from the lens; $\frac{1}{10}-\frac{1}{20}=\frac{1}{20}$.

(Dr. Giraud-Teulon, of Paris, has ascribed the origination of the above theory to Mr. J. Z. Laurence, of London, to whom we are very much indebted, for his praiseworthy efforts to popularize this, hitherto neglected, field of Physiological and Pathological Optics.)

Let me next direct your attention to certain optical considerations, which have a most important application, in the treatment of optical defects of the eye.

You may remember that in a former experiment, a 10 inch lens was held ten inches from a white wall, so as to show the miniature inverted picture of the window, &c., 20 ft. distant; and that when the lens was brought to a distance of 60 inches from the window, it was found that the image of the window was formed 12 inches behind the lens, instead of 10 inches, and that at 10 inches, the image was so indistinct as to be scarcely recognizable.

Now suppose that a 12 inch lens be immovably fixed 12 inches from the same wall, it will then be in a proper position to bring parallel rays to a focus on the wall, where it will form an inverted picture of the window, and objects at a distance beyond the window.

If we now bring the flame of a lamp, for instance, to a distance of 60 inches from the lens, no distinctly defined image of the flame will appear upon the wall; but if, by any means, we can render the pencil parallel that diverges from the flame, the 12 inch lens will then converge it accurately to a focus upon the wall, where we will have an inverted image of the flame.

From the knowledge that we have now obtained, we know that a 60 inch lens placed in front of the 12 inch lens will render these rays parallel. All that we have to do then is to combine a 60 inch lens with the 12 inch lens: the 60 inch lens to render the rays parallel that diverge from the flame, 60 inches distant, and the 12 inch lens to converge these rays to a focus, at the principal focal length of the lens. This is exactly what we do in supplying old people with convex spectacles. Their eyes are constructed to bring parallel rays to a focus, on the retina; but the rays from near objects are too divergent to be focussed upon the retina without artificial aid; this deficiency is what we supply with suitable glasses.

Before leaving the consideration of optical lenses, there is one subject to which I wish to direct your attention; namely, the formation of an inverted image behind a convex lens.

Many of you are, probably, familiar with the fact, that when light is admitted into a darkened room, through a small orifice, there appears upon the opposite wall of the room, an inverted, dim, shadowy picture of buildings, trees, &c., in front of the aperture. This can also be seen, on a smaller scale, by holding a sheet of white paper a few inches from the key-hole of a darkened hall.

The philosophy of this is seen in Fig. 5.

Let A, B, represent the position of a flame of a lamp that is a short distance in front of an aperture of a darkened box. Pencils of divergent rays of light radiate from the apex of the flame in every direction; one of these pencils is represented in the figure to illuminate the end of the box, and one of the rays escaping through the small orifice *c;* this ray passes in a straight line to the back of the box, and strikes the point *a*, which it illuminates.

Rays of light diverge from the lower part of the flame, also; one of these rays is shown to enter the aperture *c*, and to pass to the back of the box at *b*. In a similar way it might be illustrated that pencils of light radiate from every point in the flame A, B, and that one ray from each point passes into the box and illuminates a portion of the back. In this way we get an illuminated spot at the back of the box, which is an exact counterpart of the flame in front of the box, but *inverted*, the apex of the flame pointing downwards. The reason that the picture is reversed is that, as rays of light (in the same medium) pass in straight lines, a ray from the top of the flame, after passing the aperture, must necessarily pass to the lower part of the back of the box; and a ray from the lower part of the flame must necessarily (in moving in a straight line) pass to the upper part of the back of the box. You will observe, also, that the size of the image depends upon its distance behind the aperture; if the image is as far behind the aperture, as the object is in front, the image will be of the same size as the object, if half the distance, half the size, as seen at f, g.

If, in the above experiment, the aperture be enlarged, it will be found that the image at the back of the box will become much less distinct; the more the aperture is enlarged, the more indistinct will be the image. The reason of this indistinctness in the image is that, when the aperture is enlarged, a number of diverging rays from one point in the flame pass through the aperture, and each one repeats the image, so that the parts of the image overlap each other.

This is shown in Fig. 6. A, B, represents the flame of the lamp, and C, E, D, F, the image behind an aperture. The aperture is supposed to be just large enough to admit two divergent rays, each of these rays produces a separate image; thus, the point A is repeated twice at D and F, and the point B is repeated at C and E. The larger the aperture, the more light is admitted, but the more indistinct is the image.

If now, a convex lens be inserted in the enlarged aperture, these divergent rays that enter the aperture (from every point of the object) are converged to a focus; thus in

Fig. 7.

Fig. 7. A C represents an object in front of a convex lens, and *a c* the inverted image behind the lens. Rays diverging from the point A and falling upon the lens L are brought to a focus at *a*; rays from B are similarly focussed at *b*, and so on. In a similar manner, diverging rays from every point in the object A C that enter the lens are brought to a focus in the image between *a* and *c*. We will then have in the position of *a c* a distinct inverted image of the object A C. If this image is received upon a sheet of white paper we can see it only upon its front surface; but if it is received upon thin oiled paper, or upon ground glass, we can see it from behind; and if, while viewing the image from behind, the ground glass be removed, we can still see the inverted image (or at least a portion) occupying the same position as the ground glass just occupied—being suspended, as it were, in the air, and forming what is called an ærial image. In order to see this ærial image under favourable circumstances, one eye only should be used, and should be in a line with the lens and the object, and should be at least ten inches behind the position of the inverted lens.

### Chapter II.—Optics of Normal Eye.

The human eye, from before backwards, is about one inch in diameter. Its transparent media are the cornea, aqueous humour, crystaline lens, and vitreous humour. This combination, with the convexity of the cornea, is equal to a convex lens having a focus of about one inch (more accurately $\frac{18}{20}$ of an inch.)

When a normal eye is directed to a distant object (*i. e.* in a state of rest), parallel rays of light are brought to a focus upon the retina, and a very minute inverted picture of the object is sharply defined upon that membrane. If the sclerotic coat be removed from the back of the eye of an ox, and the eye be placed in an aperture of a darkened room, with the cornea looking, for instance, towards the opposite side of the street, an inverted image of the buildings, &c., in front of the aperture will be seen at the back of the eye.

The impression that objects make upon the retina, is conveyed through the optic nerve to the brain, but in what manner this communicates to the mind a knowledge of the appearance of objects, is more than we can tell. We can simply say with Potterfield, that "God has willed it so."

We are aware, however, that although the eye may be free from disease, and the connection between the retina and brain in every way perfect, if the optical mechanism of the eye be in any way defective so as to produce ill defined images upon the retina,—vision will be indistinct, and that the distinctness or indistinctness of vision will be in exact proportion to the distinctness or indistinctness of the inverted picture. Hence the necessity of understanding the optics of the eye in order to comprehend the pathology and treatment of the numerous optical defects to which it is liable.

Case 1. Let me here take an example. A few weeks ago a physician of this city sent a patient for my advice, fearing that he was losing the sight of his left eye. Upon examination, I found that he had what we call "paralysis of accommodation" of that eye.

He could see distant objects with perfect distinctness, but near objects he was unable to define; he could not read large type unless the letters were very large, and several feet from the eye. The eye was, in fact, simply passive, like a convex lens, or a camera-obscura with the screen to receive the image immovably fixed at the principal focus of the lens, and could only bring parallel rays to a focus on the retina.

I found that by rendering the diverging rays parallel, by means of a convex lens, he could see near objects distinctly; by placing a six inch convex lens before that eye, he could read fine type at six inches, with a 10 inch lens at ten inches, with an 18 inch lens at eighteen inches, &c. &c. The 6 inch lens rendered the rays parallel that diverged from the letters six inches distant, and these parallel rays falling upon the eye were brought to a focus upon the retina. [A 6 inch lens does not increase the apparent size of letters one-half, whereas this patient could not see letters ten times the ordinary size at six inches, or any distance less than about two feet from the eye.] The 10 inch lens rendered the rays parallel from objects ten inches distant, and the 18 inch lens from objects eighteen inches distant.

The eye was unable to bring diverging rays to a focus upon the retina; in other words it had lost the power of "accommodation." (We can temporarily paralyse the accommodation of the eye by applying a strong solution of Atropine.)

A normal eye differs from the glass lenses we have been describing in the fact that it can, not only focus parallel rays upon the retina, but also rays that diverge from objects as near as from four to six or eight inches from the eye. When parallel rays fall upon a 1 inch convex lens, they are brought to a focus one inch behind the lens, but if an object, for instance the flame of a lamp, be brought to within four inches of the lens, we know that the focus will fall farther than one inch behind the lens. If we wish to receive the inverted image of the lamp upon a screen, the screen must be held one inch and a third behind the lens.

Now when an object is brought to within, say four inches of the eye, it has no power to move the retina backwards to receive the image that would be formed behind that membrane, but, what answers the same purpose, it has the property of so far increasing its refractive power, as to be able not only to render parallel, these diverging rays, but also to focus them upon the retina. This increase in the power of the eye, is equal to the addition of a 4 inch lens in front of an eye that has its "accommodation" paralysed, as a 4 inch lens renders rays parallel that diverge from objects four inches distant.

FIG. 8.

Fig. 8 represents the section of a normal eye. When it is accommodated for distant objects parallel rays P, P, are focussed upon the retina at F, while diverging rays from O, would form a focus at *fd.* When, however, the eye is accommodated for the near object O, these diverging rays are focussed upon the retina at F.

The manner in which this increase in the refractive power of the eye is effected is still a disputed point. Most physiologists however are now inclined to the theory that it is caused by an increase in the curvature,—a thickening from before backwards, of the crystaline lens.*

* The accommodotion of the eye was at one time believed to be produced by the external muscles, but it is now ascertained that the accommodation can remain perfect with all the external muscles paralysed.

The iris was thought, by others, to have the power of increasing the refractive power of the eye, but it was proved by a case that occurred in Dr. Von Graefe's practice that accommodation can still be effected with entire absence of the iris.

Helmholtz and Cramer have proved by means of the opthalmometre, that when the eye is accommodated for a near object it undergoes the following changes:—

*The "near" and "far" point.*—The nearest point to which objects can be brought to an eye and be seen with perfect distinctness, is called the "near" point, and the farthest point of distinc vision is called the "far" point.

In a normal eye the "near" point is about seven inches from the front of the cornea, and the "far" point is at an unlimited distance. In childhood, however, the "near" point is about $3\frac{1}{2}$ inches from the eye and recedes as age advances. At the age of forty the "near" point of a normal eye is nearly eight inches from the eye.

When the "near" point recedes to a greater distance than eight inches from the eye it becomes inconvenient; such an eye is called *presbyopic* or long-sighted.

When the "far" point is not unlimited, but is at a definite distance from the eye, as for instance from six inches to four or five feet from the eye—such an eye is called *myopic* or short-sighted.

*Range of Accommodation.*—The distance between the "near" and "far" point in any eye, is called the "range of accommodation." If a person can read distinctly very fine type at four inches from the eye, and can also see clearly at an infinite distance the range of accommodation would be said to equal $\frac{1}{4}$ because, when such an eye is directed to objects at an infinite distance, (accommodated for parallel rays) in order to see clearly objects only four inches distant, it is necessary to increase the curvature of the crystaline lens, or in other words the "power" of the eye to an extent equal to the addition of a 4 inch convex lens; the power of which is expressed by $\frac{1}{4}$. If a person's "near" point is at eight inches from the eye, and his "far" point at an infinite distance. his range of accommodation would be said to equal $\frac{1}{8}$.

If the "near" point of a myopic eye be 3 inches, and the "far" point be 12 inches, we get the range of accommodation by the equation $\frac{1}{3}-\frac{1}{12}=\frac{1}{4}$.

---

1st. The pupil contracts; 2nd. The pupillary edge of the iris moves forward; 3rd. The peripheral portion of the iris moves backwards; 4th. The anterior surface of the lens becomes more convex (arched); 5th. The lens does not change its position; 6th. The cornea retains the same degree of curvature.

## Figures and Tables

**Fig 1 f1:**
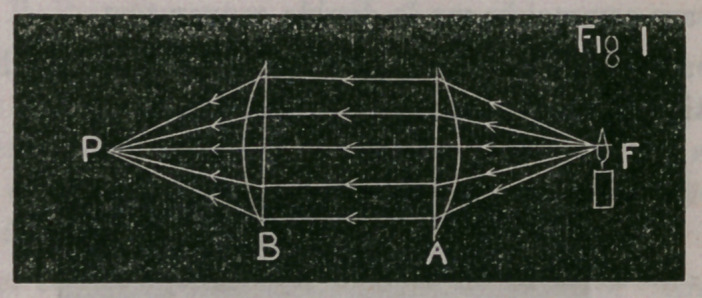


**FIG 2 f2:**
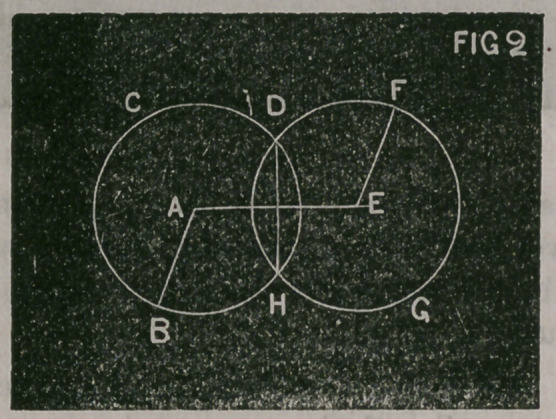


**Fig 3 f3:**
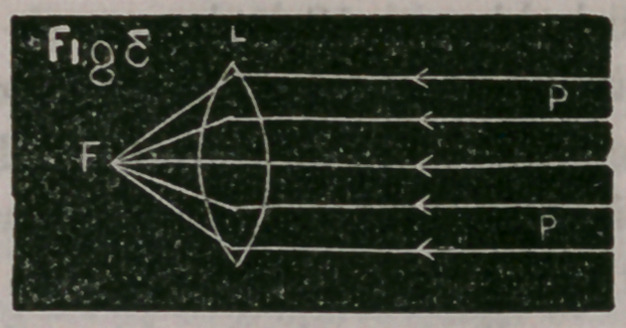


**FIG. 4. f4:**
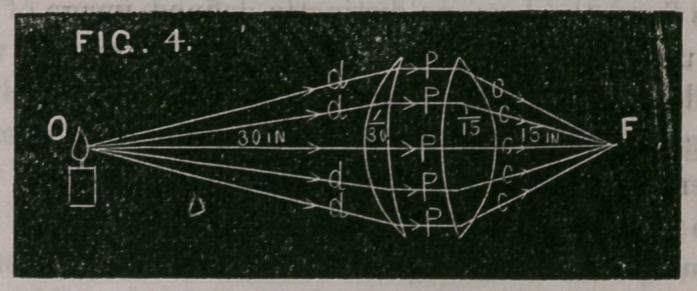


**FIG. 5. f5:**
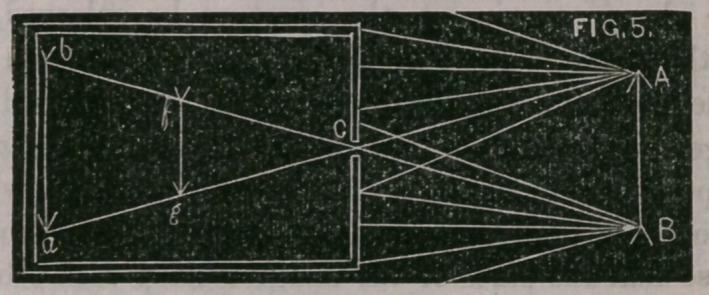


**FIG 6 f6:**
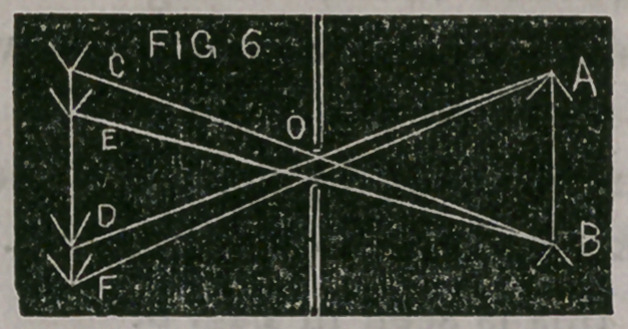


**Fig. 7. f7:**
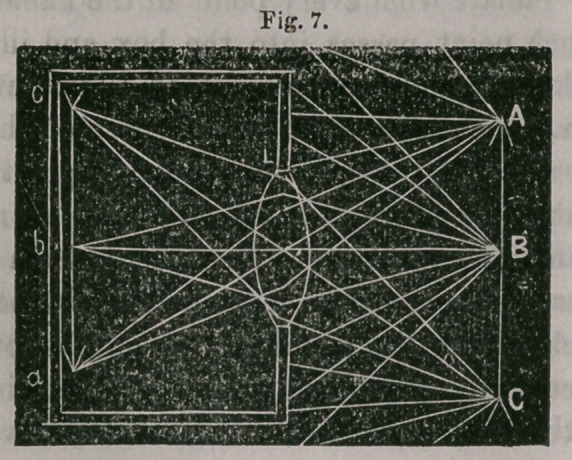


**FIG. 8. f8:**